# The comparison of the impact of arterial stiffness and central pressure on left ventricular geometry and diastolic function

**DOI:** 10.1186/s40885-019-0125-9

**Published:** 2019-09-01

**Authors:** Hack-Lyoung Kim, Woo-Hyun Lim, Jae-Bin Seo, Sang-Hyun Kim, Zoo-Hee Zo, Myung-A Kim

**Affiliations:** Division of Cardiology, Department of Internal Medicine, Seoul National University College of Medicine, Boramae Medical Center, 5 Boramae-ro, Dongjak-gu, Seoul, 07061 South Korea

**Keywords:** Arterial stiffness, Central blood pressure, Diastolic function, Left ventricle, Morphology

## Abstract

**Background:**

This study was performed to compare the associations of brachial-ankle pulse wave velocity (baPWV) and central blood pressure (CBP) measurements with left ventricular (LV) geometry and diastolic function.

**Methods:**

A total of 77 subjects (64.5 ± 10.8 years, 67.5% females) without documented cardiovascular disease were prospectively recruited. All subjects underwent transthoracic echocardiography, baPWV and noninvasive measurement of CBP on the same day.

**Results:**

In simple linear correlation analyses, neither baPWV nor CBP was associated with LV mass index or relative wall thickness (*P* > 0.05 for each). Although baPWV significantly correlated with septal e´ velocity in simple linear correlation analyses (*r* = 0.258, *P* = 0.025), the significance was lost after controlling for potential confounder (*P* = 0.881). In simple linear correlation analyses, central systolic blood pressure (CSBP) and central pulse pressure (CPP) significantly correlated with both septal e´ velocity or E/e´ (*P* <  0.05 for each); however, neither central diastolic nor mean arterial pressures was associated with both septal e´ velocity and E/e´ (*P* > 0.05 for each). After controlling for confounders, including age, sex and body mass index, CSBP correlated with septal e´ velocity (*β* = − 0.258, *P* = 0.025), but not with E/e´ (*P* = 0.074). CPP correlated with both septal e´ velocity (*β* = − 0.300, *P* = 0.014) and E/e´ (*β* = 0.428, *P* = 0.002) in the same multivariable model.

**Conclusions:**

In subjects without documented cardiovascular disease, CSBP and CPP may be more strongly associated with LV diastolic function than baPWV. Further studies with a larger sample size are needed to confirm our results.

## Background

Arteries stiffen with age, and cardiovascular diseases progress [1]. Information on arterial stiffness is clinically important in that it is associated with future cardiovascular events and mortality, independently of traditional risk factors [2–4]. Among several types of methods to measure arterial stiffness, pulse wave velocity (PWV) has been most widely used [5].

Central blood pressure (CBP) is the pressure in the ascending aorta, just outside of the left ventricle (LV). Based on the fact that major vital organs, such as the heart and the brain, are more closely exposed to CBP than to brachial blood pressure (BrBP), emerging evidence has suggested that CBP is more predictive of future cardiovascular events than BrBP [6–8]. Also, CBP has been shown to be more closely associated with subclinical target organ damage, such as left ventricular (LV) hypertrophy and carotid atherosclerosis, than BrBP [7–9]. For these reasons, CBP measurement may be valuable in clinical practice. Although an invasive hemodynamic study is the gold standard for CBP measurement, it is not feasible in routine practice due to its invasiveness, cost and time. Fortunately, radial artery applanation tonometry, a recently developed noninvasive method, showed a strong correlation with invasively measured CBP (10) and is well-validated in many studies [6, 10].

It has been suggested that there was a close interaction between the LV and the aorta [11–15]. Arterial stiffening augments central systolic and pulse pressure (CSBP and CPP, respectively) leading to LV hypertrophy and LV diastolic dysfunction. Also, decreased central diastolic blood pressure (CDBP) in a stiffened aorta reduces coronary perfusion [15]. Although both arterial stiffness and CBP have a significant impact on the LV, most of the prior studies have only focused on arterial stiffness or CBP. It is of clinical value in patient management to find out what parameter affects the LV in the same subject. However, it has not been studied yet. Therefore, this study was performed to compare the associations of brachial-ankle pulse wave velocity (baPWV) and CBP measurements with LV geometry and diastolic function.

## Methods

### Study population

Between January and March 2018, consecutive adult subjects (≥ 18 years) who visited Seoul Metropolitan Government Seoul National University Boramae Medical Center (Seoul, Korea) for cardiovascular examinations were prospectively enrolled in this study. Subjects were eligible if they had been never diagnosed with myocardial infarction, angina requiring revascularization, heart failure, peripheral arterial disease or stroke. Subjects with the following clinical conditions were also excluded: 1) unstable vital signs, 2) left ventricular ejection fraction < 50%, 3) valvular regurgitation or stenosis of mild degree and greater, 4) uncontrolled arrhythmia, and 5) ankle-brachial index < 0.9 or > 1.4. The study protocol was approved by the Institutional Review Board of Boramae Medical Center (Seoul, Korea). Written informed consent was obtained from each study subject.

### Data collection

Body mass index (BMI) was calculated by dividing body weight (kg) by the square of body height (m). Hypertension was defined as systolic blood pressure ≥ 140 mmHg, diastolic blood pressure ≥ 90 mmHg, previous diagnosis of hypertension, or the use of antihypertensive medications. Diabetes mellitus, was defined as fasting glucose ≥ 126 mg/dL, previous diagnosis of diabetes mellitus or the use of anti-diabetic medications. Dyslipidemia was defined as low-density lipoprotein cholesterol ≥ 160 mg/dL, previous diagnosis of dyslipidemia, or the use of anti-dyslipidemic medications. Subjects were classified as smokers if they had smoked within the recent 12 months. All subjects underwent laboratory tests with venous blood taken in the morning after overnight fasting. White blood cell (WBC) count and hemoglobin concentration as well as serum levels of fasting glucose, total cholesterol, low-density lipoprotein (LDL) cholesterol, high-density lipoprotein (HDL) cholesterol, triglyceride, glycated hemoglobin (HbA1c) creatinine, and C-reactive protein were measured by an automated enzymatic procedure. Estimated glomerular filtration rate was calculated using the 4-component Modification of Diet in Renal Disease (MDRD) study equation incorporating age, race, sex, and serum creatinine level [16]. Data on cardiovascular medications, including calcium channel blocker, beta-blocker, angiotensin receptor blocker and statin, was also collected.

### baPWV measurement

The volume-plethysmographic apparatus (VP-2000; Colin Co., Ltd., Komaki, Japan) was used for the measurement of baPWV. Each baPWV was measured in accordance with the manufacturer’s instructions [17, 18]. Caffeine consumption, cigarette smoking, and alcohol consumption were prohibited before baPWV measurement on the study day. Each subject rested in the supine position in a quiet room for at least 5 min before the measurement. Electrocardiographic electrodes were applied to both wrists, phonocardiographic electrodes were placed on the edge of the sternum to detect heart sounds, and pneumatic cuffs were wrapped on both upper arms and ankles. PWV was calculated by dividing distance by transit time. The distance between measurement points of baPWV was estimated by subject height. Transit time was calculated from the start point of the brachial pulse wave to the start of the ankle pulse wave. The average value of left and right baPWV measurements was used for the study. The coefficient of variance for inter-observer reliability of baPWV was 5.1% in our laboratory [19].

### CBP measurement

Immediately after baPWV measurement, CBP measurement was conducted using a commercially available radial artery applanation tonometry (HEM-9000AI; Omron Healthcare, Kyoto, Japan) [20]. Left radial artery pressure waveforms and right brachial blood pressure were simultaneously measured after resting in the sitting position for ≥ 5 min. Then, we obtained the first and second peaks of peripheral systolic pressure (SBP1 and SBP2), respectively by calibrating the radial waveform with brachial systolic blood pressure. Finally, CBP was automatically calculated with a linear equation for SBP2 [21]. Pulse pressure (PP) was defined as the difference between systolic and diastolic blood pressures (SBP and DBP, respectively).

### Transthoracic echocardiography (TTE)

TTE was performed on the same day of CBP measurement. TTE was performed using commercially available devices (Sequoia; Siemens Medical Solutions, Mountain View, CA, USA or Vivid 7; GE Medical Systems, Milwaukie, WI, USA) according to the recommendations of the current guidelines [22, 23]. LV dimensions were measured on the parasternal short-axis view using M-mode echocardiography, and LV ejection fraction was measured on the apical 4- and 2-chamber views using the biplane Simpson’s method. Relative wall thickness (RWT) was defined as 2 times posterior wall thickness divided by LV diastolic diameter, and the LV mass was calculated using a validated formula and indexed to the body surface area (LV mass index [LVMI]) [22]. All patients received pulsed wave Doppler examination at the tip of the mitral leaflets to measure peak early mitral inflow velocity during early diastole (E), late diastole (A), and deceleration time. Color-coded tissue Doppler imaging was performed on the apical 4-chamber view to calculate early velocity (e′) at the septal mitral annulus. Left atrial volume index (LAVI) was measured with the biplane method on the apical 4- and 2-chamber views at ventricular end-systolic phases [23]. LV diastolic function was assessed using abnormal cutoff values (septal e′ < 7 cm/sec and E/e′ ≥ 15) recommended by the guideline [23]. Inter-observer agreements of septal e′ and E/e′ were evaluated by Pearson’s correlation among 50 subjects. The correlation coefficients were 0.96 and 0.92 for e′ and E/e′, respectively, in our laboratory [24].

### Statistical analysis

The continuous variables are presented as mean ± SD, and categorical variables are expressed as percentages. Univariate associations between 2 parameters were assessed using simple linear regression analyses. Multivariable linear regression analysis was subsequently applied to examine independent relationships between individual parameters that had significant associations in univariate analyses. Age, sex, and BMI were adjusted during multivariable analyses. Scatter plots were used for the demonstration of linear correlations between 2 parameters. Correlations were compared using Meng’s *Z* test [25]. A *P* value of < 0.05 was considered statistically significant. All statistical analyses were conducted using SPSS 22.0 (IBM Corp., Armonk, NY, USA).

## Results

### Clinical characteristics of the study subjects

Clinical characteristics of the study subjects are presented in Table 1. Mean age was 64.5 ± 10.8 years, and 67.5% were female. The prevalence rates of hypertension, diabetes mellitus, and dyslipidemia were 48.1, 19.5, and 36.4%, respectively. Approximately one-fourth of the patients (26.0%) were current smokers. The mean value of WBC count, hemoglobin concentration, serum level of total cholesterol, LDL cholesterol, HDL cholesterol, triglyceride, HbA1c, glomerular filtration rate, and C-reactive protein were within the normal range. The proportion of subjects taking calcium channel blocker, beta-blocker, renin-angiotensin system blocker, and statin were 13.0, 16.9, 7.8, and 15.6%, respectively. Results of TTE, baPWV,. and CBP are shown in Table 2. All these parameters were within normal limits. The mean value of baPWV was 1,512 ± 299 cm/s, and the mean values of central SBP and DBP were 137 ± 19 and 78.3 ± 9.7 mmHg, respectively.

**Table 1:** Clinical characteristics of study subjects

Characteristic	Value (*n* = 77)
Age, years	64.5 ± 10.8
Female sex, *n* (%)	52 (67.5)
Body mass index, kg/m^2^	25.0 ± 3.7
Cardiovascular risk factors, *n* (%)	
Hypertension	37 (48.1)
Diabetes mellitus	15 (19.5)
Dyslipidemia	28 (36.4)
Cigarette smoking	20 (26.0)
Laboratory findings	
White blood cell count, per microliter	6,276 ± 1,772
Hemoglobin, g/dL	13.6 ± 1.8
Total cholesterol, mg/dL	174 ± 37
LDL cholesterol, mg/dL	102 ± 34
HDL cholesterol, mg/dL	52.9 ± 12.0
Triglyceride, mg/dL	121 ± 76
HbA1c, %	5.97 ± 0.98
Glomerular filtration rate, mL/min/1.73m^2^	88.2 ± 20.5
Glomerular filtration rate < 60 mL/min/1.73m^2^, *n* (%)	4 (5.2)
C-reactive protein, mg/dL	0.35 ± 0.92
Concomitant medications, *n* (%)	
Calcium channel blocker	10 (13.0)
Beta-blocker	13 (16.9)
Angiotensin receptor blocker	6 (7.8)
Statin	12 (15.6)

**Table 2:** Results of echocardiography and measurements of arterial stiffness and blood pressure

Parameter	Value (*n* = 77)
*Echocardiography*	
Left ventricular end-diastolic dimension, mm	47.8 ± 5.0
Left ventricular end-systolic dimension, mm	29.8 ± 5.8
Left ventricular ejection fraction, %	66.2 ± 5.4
Relative wall thickness	0.35 ± 0.04
Left ventricular mass index, g/m^2^	82.9 ± 21.2
E/A	0.87 ± 0.36
Deceleration time, ms	219 ± 50
Septal e’ velocity, cm/s	6.46 ± 4.31
Septal E/e’	11.4 ± 4.4
Left atrial volume index, mL/m^2^	33.1 ± 11.0
Maximal velocity of tricuspid regurgitation flow, m/s	2.24 ± 0.33
*Brachial ankle pulse wave velocity, cm/s*	1,512 ± 299
*Blood pressure measurements*	
Brachial systolic blood pressure, mmHg	131 ± 18
Brachial diastolic blood pressure, mmHg	76.1 ± 9.9
Brachial mean arterial pressure, mmHg	94.6 ± 12.0
Brachial pulse pressure, mmHg	55.6 ± 12.5
Central systolic blood pressure, mmHg	137 ± 19
Central diastolic blood pressure, mmHg	78.3 ± 9.7
Central mean arterial pressure, mmHg	98.0 ± 11.2
Central pulse pressure, mmHg	59.0 ± 15.9

### Associations of arterial stiffness and CBP with LV geometry and diastolic function

**Fig. 1:**
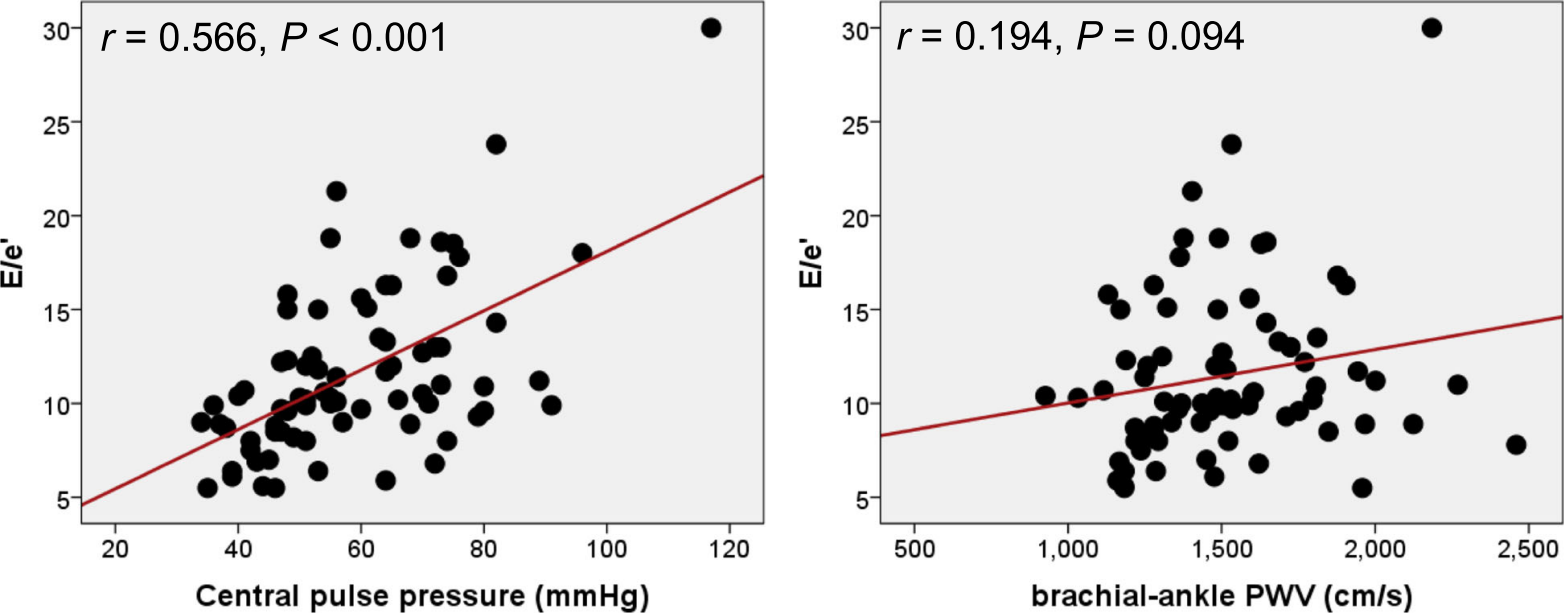
The correlations of central pulse pressure and brachial-ankle PWV with Ee/'. PWV, pulse wave velocity

**Fig. 2:**
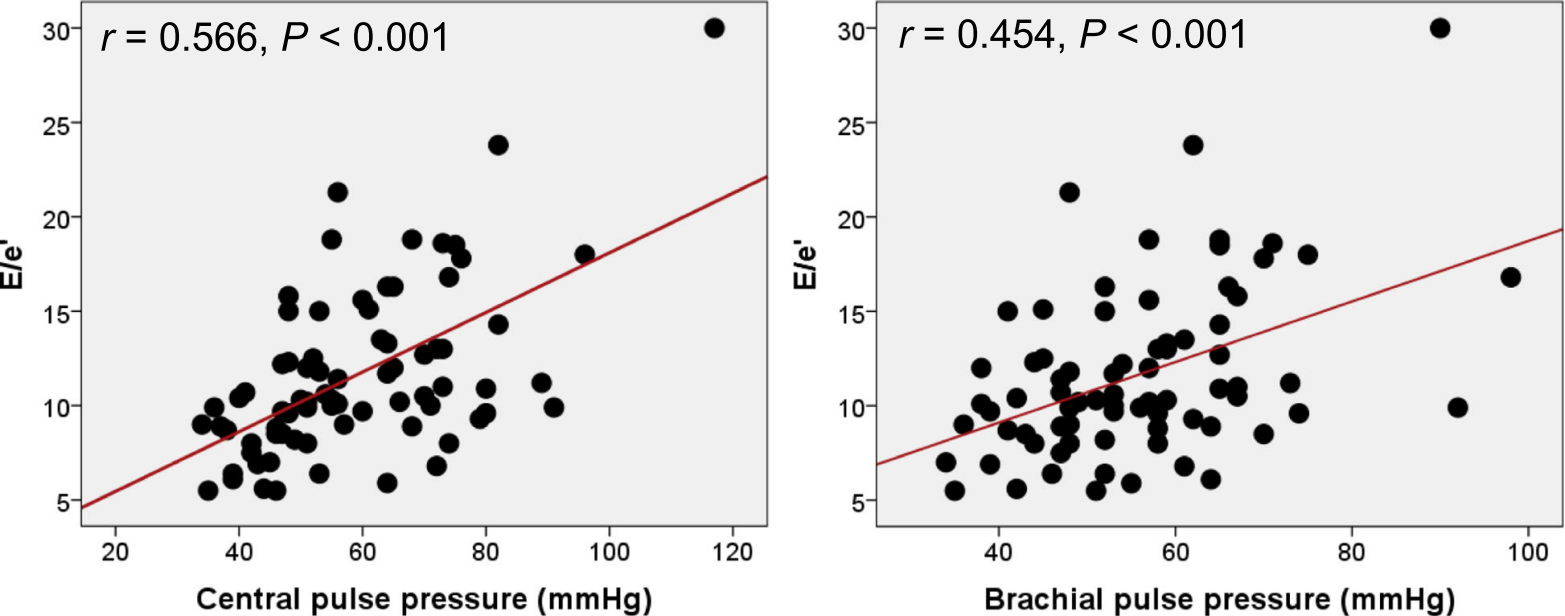
The correlations of central and brachial pulse pressures with E/e'

Univariable and multivariable analyses showing the associations of arterial stiffness and CBP with LV geometry and diastolic function are demonstrated in Table 3. The baPWV was not associated with LV geometric parameters, such as RWT or LVMI, in simple linear correlation analyses (*P* > 0.05 for each). It correlated with e′ velocity in simple correlation analysis (*r* = − 0.258, *P* = 0.025); however, the statistical significance disappeared in multivariable analysis (*P* = 0.881). CSBP correlated with e′ velocity (*r* = − 0.386, *P* = 0.001) and E/e′ (*r* = 0.314, *P* = 0.003), but not with RWT (*P* = 0.768) or LVMI (*P* = 0.869) in simple correlation analyses. In multivariable analysis after controlling for confounding effects of age, sex, and BMI, the statistical significance remained in the association between CSBP and e′ velocity (*r* = − 0.239, *P* = 0.025), but not in the association between CSBP and E/e′ (*r* = 0.212, *P* = 0.074). Neither CDBP nor CMAP was not associated with RWT, LVMI, e′ velocity, or E/e′ in simple correlation analyses (*P* > 0.05 for each). CPP correlated with e′ velocity (*r* = − 0.490, *P* <  0.001) and E/e′ (*r* = 0.566, *P* <  0.001), but not with RWT (*P* = 0.984) or LVMI (*P* = 0.333) in simple correlation analyses. The statistical significances in the associations of CPP with e′ velocity (*β* = − 0.300, *P* = 0.014) and E/e′ (*β* = 0.428, *P* = 0.002) remained even after controlling for age, sex, and BMI in multivariable analyses. The linear correlations of baPWV and CPP with E/e′ are demonstrated in Fig. 1. Brachial PP also showed a significant correlation with E/e′ (*r* = 0.454, *P* <  0.001). The correlation coefficient was numerically smaller between brachial PP and E/e′ than between CPP and E/e′, although it was statistically insignificant (*P* for difference = 0.111) (Fig. 2).

**Table 3:** Associations of brachial-ankle pulse wave velocity and central blood pressure measurements with parameters of left ventricular geometry and diastolic function

Parameter	Simple		Multivariable^a^	
	*r*	*P*	*β*	*P*
*brachial-ankle pulse wave velocity*				
Relative wall thickness	0.116	0.316	–	–
Left ventricular mass index	−0.071	0.547	–	–
e´ velocity	−0.258	0.025	− 0.017	0.881
E/e´	0.194	0.094	–	–
*Central systolic blood pressure*				
Relative wall thickness	0.034	0.768	–	–
Left ventricular mass index	0.019	0.869	–	–
e´ velocity	−0.386	0.001	−0.239	0.025
E/e´	0.341	0.003	0.212	0.074
*Central diastolic blood pressure*				
Relative wall thickness	0.063	0.588	–	–
Left ventricular mass index	−0.135	0.250	–	–
e´ velocity	0.054	0.640	–	–
E/e´	−0.161	0.101	–	–
*Central mean arterial pressure*				
Relative wall thickness	0.055	0.633	–	–
Left ventricular mass index	−0.069	0.558	–	–
e´ velocity	−0.187	0.105	–	–
E/e´	0.101	0.385	–	–
*Central pulse pressure*				
Relative wall thickness	0.002	0.984	–	–
Left ventricular mass index	0.113	0.333	–	–
e´ velocity	−0.490	< 0.001	−0.300	0.014
E/e´	0.566	< 0.001	0.428	0.002

^a^ Age, sex and body mass index are adjusted in this model

## Discussion

The main findings of the present study are: 1) there were lack of associations of both baPWV and CBP with LV geometry; and 2) CSBP and CPP, but not baPWV, were associated with LV diastolic function. To the best of our knowledge, this is the first report showing more powerful association of CBP with LV diastolic function compared to arterial stiffness in the same subject.

### Association between CBP and LV diastolic function

The association between CBP and LV diastolic function has been reported. Previously, our group has reported a significant association between invasively measured pulsatile pressure of the central aorta and LV diastolic parameters in 153 patients undergoing invasive coronary angiography [24]. Shim et al. [11] measured CBP using radial artery tonometry and showed that CPP amplification was independently associated with e′ velocity in women. A study of 1,233 community subjects demonstrated that CPP was associated with e′ velocity and E/e′ ratio [13]. A recent longitudinal study of 607 subjects found that the increased E/e′ ratio during the 4.7 years of follow-up was independently related to baseline CPP measured by radial artery tonometry in women [26]. In line with these studies, our study showed the association of CSBP and CPP with e′ velocity, which supports the concept of ventricular-arterial relationship [15, 27, 28]. Increased CSBP represents increased LV afterload, which may slow LV relaxation. Additionally, widening of CPP with decreased CDBP reduces coronary perfusion during LV diastole, causing further impairment of myocardial relaxation [15, 29].

### Lack of the association between baPWV and LV diastolic function

There have been several studies reporting the association of arterial stiffness measured by baPWV with LV diastolic function. Analysis of 320 subjects with various abnormalities of cardiac structure and function have suggested that baPWV correlates with parameters of LV structure and function such as LV mass and mitral e′ velocity [30]. Wang et al. [31] reported that baPWV was independently and negatively associated with e′ velocity in 42 hypertensive subjects. In apparently healthy 115 subjects, there was a significant correlation between baPWV and E/e′ [32]. Similarly, Kang et al. [33] investigated 2,095 community subjects and showed an independent association between baPWV and LV diastolic dysfunction. A previous study confirmed that baPWV was significantly associated with E/A ratio among healthy adults with normal LV ejection fraction [34]. On the contrary, the results of the present study showed that baPWV correlated with septal e′ velocity in univariable analyses; however, the significance of this correlation disappeared after controlling for age, sex, and BMI. It is thought that a different study population and a small sample size might be the main reason for the negative findings of our study. Further studies with a larger sample size are needed to determine the utility of baPWV and CBP in the prediction of LV diastolic function.

### Clinical implications

A novel finding of our study is that CBP more affects LV diastolic function than arterial stiffness. This may suggest the importance of CBP as an indicator of LV diastolic dysfunction, which provides an additional insight in the interaction between LV and the arterial system. Given that LV diastolic dysfunction is associated with worse clinical outcomes [35, 36], CBP measurement may have more important implications for risk stratification than baPWV. CBP may be a good monitoring tool to assess LV diastolic dysfunction. In addition, revealing the degree of relationships between baPWV and CBP may be particularly important for treatment strategies. Targeting CBP may be more effective in reduction in patient risk than targeting baPWV. The potential targeted therapy should be explored to reduce CBP for the restoration of LV diastolic dysfunction or the prevention of heart failure. Moreover, there is lack of corroborative evidence that CBP reduction leads to the improvement in cardiovascular outcomes. Further prospective studies are needed to confirm this.

### Study limitations

There are several limitations to this study. First, it is difficult to prove the causal relationship between CBP and LV diastolic function in this cross-sectional study. Secondly, there was the possibility that the small sample size of our study could not draw statistical significance in the relationships between baPWV and LV diastolic parameters, and between baPWV/CBP and LV geometry. For the same reason, some potential confounders, such as medications, could not be controlled during multivariable analyses. Also, although sex differences in ventricular-arterial relationships have been an issue [11, 26], sex-specific analysis could not be performed due to the small number of study subjects. Thirdly, baPWV was used as a measure of arterial stiffness in our study, instead of carotid-femoral PWV (cfPWV), a gold standard noninvasive measure of arterial stiffness [37]. However, baPWV has been shown to be strongly associated with cfPWV [38] and invasively measured aortic PWV [39]. In some studies, baPWV better correlated with LV mass and diastolic function than cfPWV [30]. Since baPWV includes both central and peripheral arterial stiffness, and cfPWV mainly reflects only central arterial stiffness, baPWV may be more representative of an arterial load of the LV than cfPWV [40]. Therefore, baPWV can be used as an indicator of arterial stiffness. Finally, our study subjects were middle-aged or older and without overt cardiovascular disease, so that our results cannot be generalized to other groups of subjects with different demographics and risk factors.

## Conclusions

In middle-aged or older subjects without overt cardiovascular disease, CSBP and CPP were more strongly associated with LV diastolic function than baPWV. Measurement of CBP rather than baPWV may be more useful for the risk stratification and management of these age groups of subjects. Further studies with a larger sample size are warranted.

## Data Availability

The datasets used and/or analyzed during the current study are available from the corresponding author on reasonable request.

## Abbreviations

baPWV: brachial-ankle pulse wave velocity
BMI: Body mass index
BrBP: Brachial blood pressure
CBP: Central blood pressure
CDBP: Central diastolic blood pressure
cfPWV: carotid-femoral pulse wave velocity
CPP: Central pulse pressure
CSBP: Central systolic blood pressure
DBP: Diastolic blood pressure
HDL: High-density lipoprotein
LAVI: Left atrial volume index
LDL: Low-density lipoprotein
LV: Left ventricle
LVMI: Left ventricular mass index
MDRD: Modification of Diet in Renal Disease
PP: Pulse pressure
PWV: Pulse wave velocity
RWT: Relative wall thickness
SBP: Systolic blood pressure
TTE: Transthoracic echocardiography
WBC: White blood cell
